# *OsLSC6* Regulates Leaf Sheath Color and Cold Tolerance in Rice Revealed by Metabolite Genome Wide Association Study

**DOI:** 10.1186/s12284-024-00713-z

**Published:** 2024-05-13

**Authors:** Shuwei Lv, Xuan Tang, Liqun Jiang, Jing Zhang, Bingrui Sun, Qing Liu, Xingxue Mao, Hang Yu, Pingli Chen, Wenfeng Chen, Zhilan Fan, Chen Li

**Affiliations:** Rice Research Institute, Guangdong Academy of Agricultural Sciences, Guangdong Key Laboratory of New Technology in Rice Breeding, Guangdong Rice Engineering Laboratory, Key Laboratory of Genetics and Breeding of High Quality Rice in Southern China (Co-Construction By Ministry and Province), Ministry of Agriculture and Rural Affairs, Guangzhou, 510640 China

**Keywords:** Rice, mGWAS, *OsLSC6*, Anthocyanin biosynthesis, Cold tolerance

## Abstract

Plant metabolites including anthocyanins play an important role in the growth of plants, as well as in regulating biotic and abiotic stress responses to the environment. Here we report comprehensive profiling of 3315 metabolites and a further metabolic-based genome-wide association study (mGWAS) based on 292,485 SNPs obtained from 311 rice accessions, including 160 wild and 151 cultivars. We identified hundreds of common variants affecting a large number of secondary metabolites with large effects at high throughput. Finally, we identified a novel gene namely *OsLSC6* (*Oryza sativa* leaf sheath color 6), which encoded a UDP 3-*O*-glucosyltransferase and involved in the anthocyanin biosynthesis of Cyanidin-3-Galc (sd1825) responsible for leaf sheath color, and resulted in significant different accumulation of sd1825 between wild (purple) and cultivars (green). The results of knockout transgenic experiments showed that *OsLSC6* regulated the biosynthesis and accumulation of sd1825, controlled the purple leaf sheath. Our further research revealed that *OsLSC6* also confers resistance to cold stress during the seedling stage in rice. And we identified that a SNP in *OsLSC6* was responsible for the leaf sheath color and chilling tolerance, supporting the importance of *OsLSC6* in plant adaption. Our study could not only demonstrate that* OsLSC6* is a vital regulator during anthocyanin biosynthesis and abiotic stress responses, but also provide a powerful complementary tool based on metabolites-to-genes analysis by mGWAS for functional gene identification andpromising candidate in future rice breeding and improvement.

**Supplementary Information:**

The online version contains supplementary material available at 10.1186/s12284-024-00713-z.

## Introduction

Plants produce a variety of metabolites selected by environment and human beings are important for both their lives and human health (Schauer et al. 2006; Cao et al. 2022). Metabolomics, serve as the intermediate and ultimate products of biological processes, are invaluable for both phenotyping and diagnostic studies in plants and humans (Kettunen et al. 2012; Griffin 2006; Fernie and Schauer 2009). Meanwhile, metabolic phenotype builds a bridge between genes and visible phenotypes, which can be used as biomarkers for crop trait prediction (Matsuda et al. 2012; Riedelsheimer et al. 2012; Hirai et al. 2007). Understanding the genetic basis of natural variations in the metabolome of major crops such as rice, is important for the quality, reliability and sustainability of the world’s food supply.

Anthocyanins, belonging to the flavonoid pigment molecules (Reddy et al. 1995), are second largest metabolites which are widely distributed in different organs and tissues such as roots, leaves and flowers, responsible for the purple, red, blue and orange colors and play a crucial role in attracting pollinators (Miller et al. 2011). Meanwhile, anthocyanins also play a key role in resisting biotic and abiotic stresses, such as cold, drought, ultraviolet rays and pest diseases (Dixon et al. 2002; Hichri et al. 2011; Isshiki et al. 2014; Schulz et al. 2015, 2016; Zhang et al. 2020). For example, overexpression of *UGT79B2* and *UGT79B3* in *Arabidopsis* significantly enhanced plant tolerance to cold stress, primarily due to increased the anthocyanin accumulation (Li et al. 2017a, b). Moreover, as a plant phytonutrient, anthocyanins have strong anti-mutation and antioxidant activities, and are important to human health, result in a high economic value (Li et al. 2017a, b). For the reason of their diversity and importance, anthocyanins have become one of the most studied metabolic in plants (Petroni et al. 2011; Sobel et al. 2013).

A large number of genes encode various enzymes in plants, and catalyze the biosynthesis of pro-anthocyanidins (PAs) and anthocyanins, subsequently transported to vacuoles for storage through various modifications (Tanaka et al. 2008; Zhao and Dixon 2010; Gomez et al. 2011). The genes involving anthocyanins biosynthesis could divided into two groups, named early (EBGs) and late (LBGs) biosynthetic genes (Pelletier et al. 1999; Lepiniec et al. 2006), which encode multiple enzymes that synthesize PAs and anthocyanins (Takashi et al. 2014), and regulated by MBW protein complex, so called MYB-bHLH (basic helix-loop-helix transcription factor)-WD40 repeat protein (WDR) (Winkel-Shirley 2001; Baudry et al. 2004; Xu et al. 2015). Wild rice accumulate anthocyanin in many tissues, but these flavonoid pigments are absent in most cultivars possibly as a result of artificial selection (Zheng et al. 2019). Five putative regulators of anthocyanidin biosynthesis were isolated and characterized in rice, including the R2R3-MYB transcription factor gene *OsC1* and four bHLH genes, *Ra1*/*OsB1*, *Rb*, *Ra2* and *OsB2* (Reddy et al. 1998; Sakamoto et al. 2001; Saitoh et al. 2004). The gene functional analysis showed that *OsC1* is the determinant factor of anthocyanin biosynthesis in rice leaf sheath (Chin et al. 2016), which homologous to the maize anthocyanin biosynthesis gene *C1* (Cone et al. 1986), and believed a domestication related gene caused the loss of anthocyanin accumulation in cultivars (Huang et al. 2010). Recently, the *C*-*S*-*A* gene regulation model (*OsC1*-*OsB2*-*OsDFR*) has been proposed, which regulates anthocyanin pigmentation and reveals the evolution of anthocyanin biosynthesis pathway in rice hull (Sun et al. 2018). The WD40 repeat gene *OsTTG1* is a vital regulator of anthocyanin biosynthesis in rice, phylogenetic analysis showed that directional selection has drove the divergence of *OsTTG1* alleles between *indica* and *japonica* rice (Yang et al. 2021). 

Glycosylation of anthocyanidin is usually catalyzed by UDP dependent glycosyltransferases (UGTs) (Bowles et al. 2006), and UGTs play a crucial role in regulating the endogenous balance and biological activity of anthocyanins, consequently affecting plant metabolic stress tolerance (Li et al. 2017a, b; Rao et al. 2019). Up to now, some UGTs have been found, and validated to play a positive role in enhancing rice tolerance to many abiotic stresses, including salt, drought, cold, high temperatures and UV-B irradiation (Shi et al. 2020; Liu et al. 2021; Wang et al. 2023). Despite the identification of these UGTs as key players in rice responses to abiotic stresses, the comprehensive biological function of these UGTs are largely unknown.

Cultivated rice is one of the earliest domesticated crops, and provides necessary nutrients to humans (Chen et al. 2014). The genus *Oryza* comprises wild and domesticated species makes a comprehensive metabolomic study of this species imperative. Wild and cultivated rice showed significant differences in anthocyanin biosynthesis in leaf (Zheng et al. 2019), resulted purple leaf sheath was common in wild rice, and rarely seen in cultivars. So far, only a few genes such as *OsC1* directly conferring biosynthesis of anthocyanin in purple leaf sheath were isolated (Chin et al. 2016), the biosynthesis of anthocyanin and the mechanism of this biosynthesis difference have not been widely studied, and how natural or artificial selection has reshaped the metabolite profiles of leaf sheath color remain largely unknown.

## Results

### Metabolic Profiling of Wild and Cultivated Rice Accessions

**Fig. 1:**
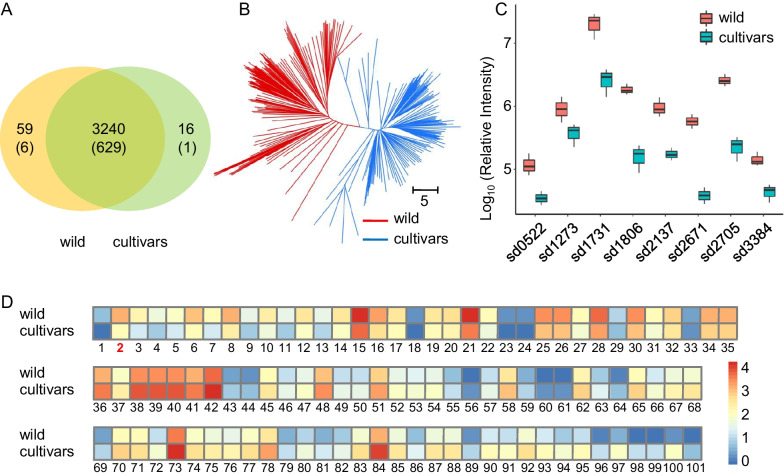
Comparison of metabolic profiles in the wild rice and cultivars. **A** Metabolic features detected in wild rice and cultivars. Number of annotated metabolites is indicated in the bracket. **B** Neighbor-jointing phylogenetic tree constructed using metabolites identified in 311 rice accessions. Two major clades, represented by wild and cultivars, could identified from the phylogenetic tree. The scale bar indicates the simple matching distance. **C** Boxplot showing the content of alkaloids (sd1731, Trigonelline), amino acid (sd1273, N-acetylglycine), flavonoids (sd1806, Cyanidin 3-*O*-rutinoside (Keracyanin)), lipids (sd3384, 13-HOTrE(r)), nucleotides (sd0522, β-Nicotinamide mononucleotide), organic acids (sd2705, Chlorogenic acid methyl ester) and phenolic acids (sd2671, *O*-p-Coumaroyl quinic acid *O*-rutinoside derivative) in wild and cultivars. **D** Heatmap of all the 101 annotated flavonoids detected in this study. The relative values of flavonoids content in wild rice was scaled to the cultivated varieties group for each metabolite. Numbers 1 to 101 correspond to the 1st to 101st flavonoids in Table S4

To investigate the effects of numerous structurally metabolites on rice growth and development, we detected and quantified 3315 distinct metabolite features by a broadly targeted liquid chromatography-tandem mass spectrometry (LC–MS) based metabolic profiling method (Chen et al. 2013) in the leaves from 311 rice accessions, including 160 wild and 151 cultivars (Table S1). Of the detected metabolic features, including 3299 in wild (635 annotated) and 3256 in cultivars (630 annotated), with 3240 (629 annotated) were detected in both wild and cultivars (Fig. 1A). Subsequently, we identified differential metabolites features between wild and cultivars according to FC (fold change) ≥ 2 or ≤ 0.5 and VIP (variable importance in the projection) ≥ 1. Finally, of the 636 metabolites annotated with associated chemical structures, 170 differential metabolites were identified when comparing wild and cultivars. The features including mainly alkaloids (21), amino acids (15), flavonoids (76), lipids (1), nucleotides (7), organic acids (17), phenolic acids (14) and others (19) (Figure S1A). Compared with wild rice, some metabolic features changed dramatically in cultivars, for example, some flavonoids (Cyanidin 3-*O*-rutinoside and Apigenin 8-*C*-pentoside) were decreased and increased by more than 11 and 50 times, respectively (Table S2). We additionally observed that more than 66% (2174 of 3315) of the metabolic features showed the coefficient of variations (CVs) greater than 50% (Figure S1B), and the broad-sense heritability (*H*^*2*^) revealed that 66.4% of the metabolites displayed values greater than 0.5 (Figure S1C). Of these metabolic features, lignans and coumarins as well as alkaloids showed the highest CVs with average up to 200.9% and 117.36%, ranging from 11.61% (Proline betaine) to 1545.93% (Feruloylcholine) and 16.82% (Tricin 7-*O*-hexoside) to 1037.48% (Chrysin *O*-malonylhexoside), respectively. While carbohydrates displayed the lowest CVs with an average of 37.06% (Table S3), suggesting significant variation of metabolites in these materials between wild and cultivars.

The clustering analysis based on the levels of metabolic features could divided the 311 rice varieties into two groups, represented by wild and cultivars (Fig. 1B). Unsurprisingly, these two subgroups almost consistent with the neighbor-joining tree showed two divergent groups which constructed by 292,485 SNPs of sequencing the 311 accessions (Figure S1D). A principal component analysis (PCA) based on the levels of all the detected metabolites data revealed that the 311 rice accessions basically formed two distinct clusters, wild and cultivars (Figure S2), which consistent with the neighbor-jointing phylogenetic tree constructed using metabolites, indicating dynamic changes in metabolites profiles during the domestication from wild to cultivars.

Compared with cultivars, we discovered that 117 annotated metabolites features were accumulated to significantly higher levels in wild rice, including 18 alkaloids, 2 amino acids, 35 flavonoids, 7 lipids, 2 nucleotides, 16 organic acids, 13 phenolic acids, and 24 others metabolites (Table S2). Among these metabolites, for example, some representative metabolites like Trigonelline (sd1731), Cyanidin 3-*O*-rutinoside (sd1806), Chlorogenic acid methyl ester (sd2705) and 3-Methyl-1-pentanol (sd2137) showed significantly elevated levels in wild when compared with cultivars, with average levels up to 7.73-, 11.11-,11.18- and 5.63- fold, respectively (Fig. 1C, Table S2). Amino acids, such as N-acetylglycine (sd1273), lipids, such as 13-HOTrE(r) (sd3384), nucleotides, such as β-nicotinamide mononucleotide (sd0522), and phenolic acids, like *O*-p-coumaroyl quinic acid *O*-rutinoside derivative (sd2671), also displayed a higher accumulation in wild rice (Fig. 1C, Table S2), indicating high natural variability between wild and cultivars. Altogether, among the eight classes of annotated metabolites, flavonoids are obviously selected during rice domestication.

Among all the metabolites, flavonoids were the one of the most critical secondary metabolite groups because they had the largest number in all samples, indicating they are widely distributed and play a important function in rice. We found that 66 of the 101 annotated flavonoids had higher levels in cultivars than in wild rice, indicating strong positive selection of flavonoids during rice domestication, and the remaining 35 flavonoids had decline in the cultivars (Fig. 1D, Table S4). These metabolites included several color related components such as anthocyanins. Interestingly, further observation revealed that among the 66 flavonoids had higher levels in cultivars, 45.5% (30 of 66) were oxygen-decorated, and 37.9% (25 of 66) were carbon decorated. While the remaining 35 flavonoids had higher levels in wild rice, the percent of oxygen decorated flavonoids sharply increased to 71.4% (25 of 35) and the carbon-decorated flavonoids decreased to 14.3% (5 of 35) (Fig. 1D, Table S4), indicating that the oxygen-/carbon-modification of flavonoids had a dramatic change during rice domestication from wild to cultivars.

### Genetic Basis of Natural Variation in Anthocyanidins Revealed by mGWAS

**Fig. 2:**
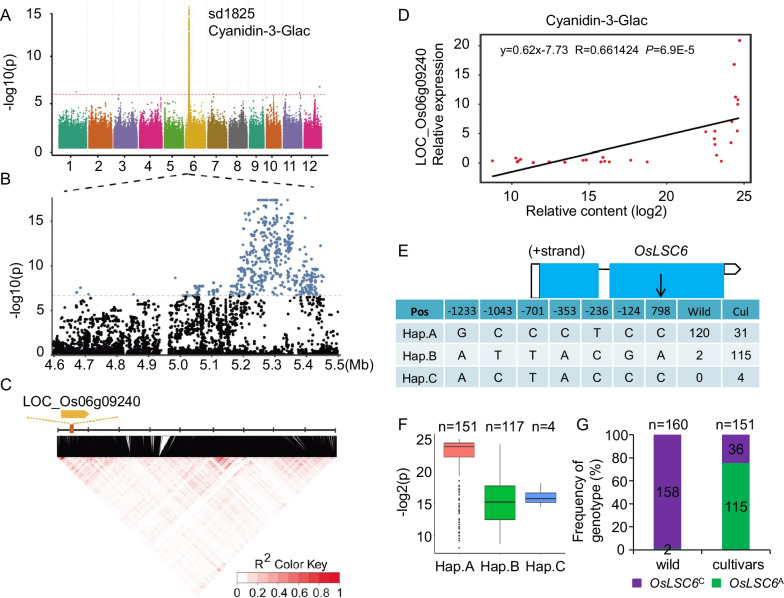
Candidate gene analysis of natural variations in sd1825 by mGWAS. **A** Manhattan plot displaying the mGWAS result of sd1825 content. **B** Part of the Manhattan plot. The threshold was set to *P* = 2.61E−7. **C** LD values among all SNPs in *OsLSC6*. The redness of the color of each box corresponds to the R^2^ value on the basis of the legend. **D** Correlation between the transcription levels of *OsLSC6* and relative content of sd1825 in 30 rice accessions. **E** The gene structure of *OsLSC6* and the number of variations in the promoter region and protein coding sequence in each haplotype. Blue boxes indicate the coding regions, black line indicate the intron, and the white boxes show the 5’ and 3’ UTRs. **F** The effect of different haplotypes on the content of sd1825. Accessions with genotype of Hap.A showed much higher sd1825 content compared with the accessions with genotype of Hap.B and Hap.C. **G** The frequency of genotypes *OsLSC6*^A^ and *OsLSC6*^C^ in 160 wild and 151 cultivars

To investigate the genetic control of the natural variation in anthocyanidins content, GWAS and mGWAS were performed by a gene-based analysis for the 311 rice accessions. Finally, one QTL distributed on Chr6 well known as *OsC1* associated with leaf sheath color was identified in our previous study (Jiang et al. 2024). Besides that, another QTL named *qLSC*6 also significantly associated with leaf sheath color was identified distributed on Chr6 in the same interval (*P*-value = 1.71E−18) (Figure S3). Meanwhile, to assist in the identification of the candidate genes to reveal the *qLSC*6, mGWAS was performed for the metabolites of anthocyanidin. Coincidentally, among the previous mentioned 101 flavonoids, we found the metabolite Cyanidin-3-Galc (sd1825, *P*-value = 4.63E−18, Figure S4A) was mainly located the same interval on Chr6, and the results of the mGWAS for sd1825 were visualized in Manhattan plots (Fig. 2A, B). Cyanidin-3-Galc was a kind of flavonoid anthocyanins, with much higher content in wild rice compared to cultivars (Figure S4B). By gene annotation and linkage disequilibrium (LD) analyses, LOC_Os06g09240, named *OsLSC6* hereafter, which encoded an anthocyanidin UDP 3-O-glucosyltransferase, was found remarkably relevant to the content of sd1825 within the confidence interval of the locus (Fig. 2C).

To further verify that *OsLSC6* is indeed the candidate gene conferred the content of sd1825, we then randomly selected the leaves of 15 wild rice accessions had higher level of sd1825 with purple leaf sheath color, and 15 cultivars had lower level of sd1825 with green leaf sheath color to perform transcriptome analysis. We measured the transcription levels of *OsLSC6*, as quantified by real-time quantitative reverse transcription PCR (qRT-PCR) across above the 30 rice varieties. The results showed that a strong correlation between the transcription levels of *OsLSC6* and sd1825 content, as confirmed by correlation analysis (Student’s *t* test, *P* < 0.05; Fig. 2D). Sequence analysis showed that there were 6 SNP variations in the promoter region and one allelic mutation (C798A) in the *OsLSC6* coding region resulted in significant change in amino acid (His-Gln) between wild and cultivars, we categorized the genotypes of *OsLSC6* into three haplotypes (Hap.A, Hap.B, Hap.C) based on these 7 SNPs (Fig. 2E). This categorization showed a significant association between these haplotypes and sd1825 content (Fig. 2F). And the SNP located within 798 bpbp of *OsLSC6* showed the most significant relevant to the levels of sd1825, with *P* values up to 6.9E−5, namely, accessions with the *OsLSC6*^C^ haplotype (Hap.A) exhibited higher sd1825 content than those with *OsLSC6*^A^ (Hap.B) (Fig. 2F). And in the 160 wild accessions, only two samples carried with the *OsLSC6*^A^ haplotype, while in the 151 cultivars, 115 carried with the *OsLSC6*^A^ haplotype (Fig. 2G, Table S5), showed that the allelic mutation (C798A) located in the *OsLSC6* coding region maybe selected during rice domestication. Taken together, these results suggest that the variation in *OsLSC6* was remarkably relevant to the content of sd1825 and influence the anthocyanin biosynthesis in rice.

### *OsLSC6* Contribute to Leaf Sheath Color and Cold Stress Tolerance

**Fig. 3:**
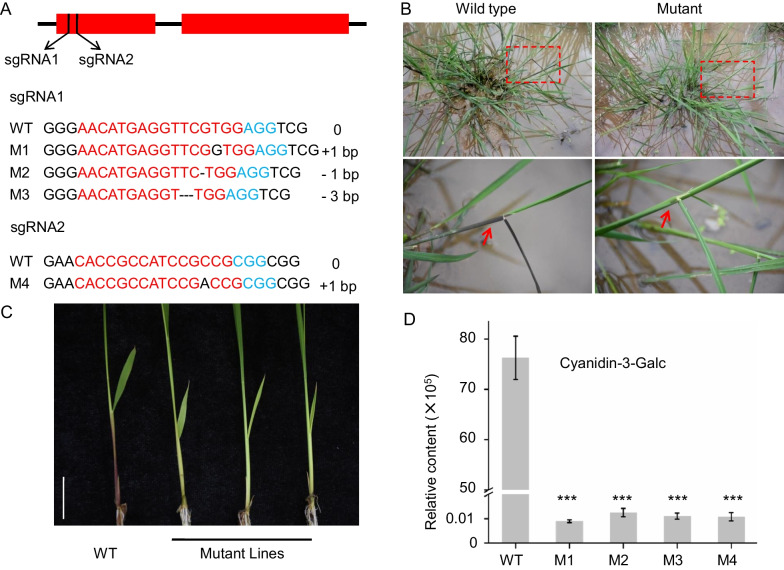
Knockout of *OsLSC6* reduces sd1825 content in rice. **A** Generation of *OsLSC6* mutation lines by CRISPR/Cas9 system. Sequences of *OsLSC6* mutant M1-M4 are shown. sgRNA targets and a protospacer-adjacent motif (PAM) are indicated in red and in blue, respectively. Deletions and insertions are indicated by dashes and in black, respectively. **B**, **C** Leaf sheath color of wild-type and *OsLSC6* mutant lines in tillering stage (B) and seedling stage (C). Red arrowheads indicated the leaf sheath, scale bar = 1 cm. **D** sd1571 content in wild-type and *OsLSC6* mutant lines. The values represent the mean ± SD of three biological replicates. The asterisks indicate significant differences between WT and *OsLSC6* mutant lines (Dunnett’s test, **P < 0.01)

*OsLSC6* was annotated as an anthocyanidin 3-*O*-glucosyltransferase, was associated with the natural variation of sd1825 which may play an important role in the formation of rice leaf color. Next, we investigated whether the *OsLSC6* gene is directly or indirectly regulated the sd1825 content, and is therefore responsible for the observed metabolic phenotype of the leaf sheath color, two CRISPR/ Cas9 constructs with different single-guide RNAs (sgRNAs) were created and transformed into the purple wild rice accession DX386. Four independent homozygous transformed lines with different mutations were obtained (Fig. 3A). In these plants, the green leaf sheath color phenotype indicates that knockout of *OsLSC6* was achieved, which strongly suggest the involvement of *OsLSC6* in the biosynthesis of the purple leaf sheath color (Fig. 3B, C).

To further validate our hypothesis that *OsLSC6* affect the content of sd1825 in rice, levels of sd1825 in the leaves of the *OsLSC6* transgenic lines were analyzed and compared with those in wild type plants by HPLC/MS. The results showed that knocking down *OsLSC6* is accompanied by a significant decline in sd1825 levels (Fig. 3D). Together, these data showed that *OsLSC6* played a role in leaf sd1825 content and responsible for the rice leaf sheath color.

**Fig. 4:**
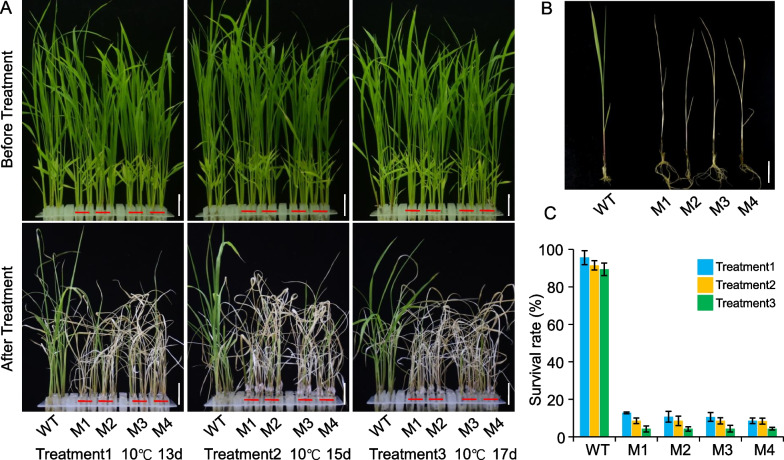
Knockout of *OsLSC6* reduces cold tress tolerance in rice. **A**, **B** Phenotypes of the WT and *OsLSC6* mutant lines before and after cold stress under three low temperature treatments. Scale bar = 2 cm. **C** Survival rates of the WT and *OsLSC6* mutant lines after cold stress under three low temperature treatments

Anthocyanins also play a crucial role in resistance to biotic and abiotic stresses, such as cold stress (Schulz et al. 2015; Li et al. 2017a, b), it appears reasonable to indicate that sd1825 is related to the adaptation of rice to chilling tolerance. To further confirm the involvement of *OsLSC6* in cold stress tolerance in rice, we subjected the seedlings of two-week-old of wild type (WT) and knockout lines (M1 to M4) to three low temperature treatments in 10℃ for 13 days, 15 days and 17 days, respectively, and followed by a 10-day recovery under normal conditions. The results clearly showed that the survival rates of WT was significantly higher than those of the knockout plants (Fig. 4). These results demonstrated that the knockout of *OsLSC6* reduces cold stress tolerance in rice. In conclusion, *OsLSC6* contribute to not only purple leaf sheath color but also cold stress tolerance in rice.

### A SNP in the Coding Region of *OsLSC6* May Confer Leaf Sheath Color and Cold Stress Tolerance in Rice

To identify the distribution of the SNP (C798A) located in the *OsLSC6* coding region in rice populations, we screened the 311 rice accessions used in this study and found that all most of the wild rice (158 of 160) harbored the *OsLSC6*^C^ haplotype, and in the 158 wild rice accessions harbored the *OsLSC6*^C^ haplotype, 142 performed purple leaf sheath color, whereas 76% (115 of 151) cultivars harbored the *OsLSC6*^A^ haplotype, and in the 115 cultivars harbored the *OsLSC6*^A^ haplotype, 101 performed green leaf sheath color (Fig. 2G and Table S5), no other allele was found. These results indicated that the SNP was associated with purple leaf sheath color in rice.

**Fig. 5:**
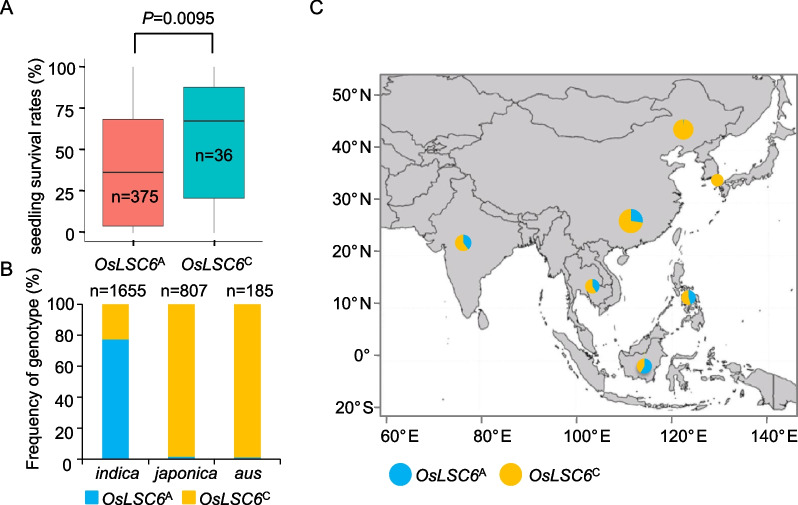
A SNP in the *OsLSC6* coding region increases rice cold tolerance. **A** 500 cultivars (including the 151 cultivars used in this study) were used for comparing the rice cold stress tolerance between the cultivars with the genotype *OsLSC6*^A^ and those with the genotype *OsLSC6*^C^. **B** The frequency of genotypes *OsLSC6*^A^ and *OsLSC6*^C^ in *indica*, *japonica* and *aus* rice. **C** The geographical distribution of cultivars with the SNP in areas of Asia

To investigate the relationship between the SNP and rice cold stress tolerance, we analyzed 411 cultivated rice accessions (including the 120 cultivars used in this study) with respect to the genotype and phenotype at the *OsLSC6* locus. Analysis showed that the 36 cultivars with the genotype *OsLSC6*^C^ displayed significantly higher seedling survival rates than the 375 cultivars with the genotype *OsLSC6*^A^ after cold treatment (Fig. 5A and Table S6). We further analyzed the distribution of the SNP in 2382 cultivated rice varieties from the 3 K RG (RFGB v2.0 database, https://www.rmbreeding.cn/). The data showed that the frequency of cultivars with the genotype *OsLSC6*^C^ in *indica*, *japonica*, and *aus* rice was 22.7, 98.4, and 98.9%, respectively (Fig. 5B), with the *japonica* varieties with *OsLSC6*^C^ being distributed mainly in East Asia, whereas the *indica* varieties with *OsLSC6*^A^ were mainly distributed in Southeast Asia (Fig. 5C). These results indicated that the SNP in the *OsLSC6* coding region confers cold stress tolerance in rice and the SNP was subject to strong selection during rice domestication.

## Discussion

Metabolites play essential roles in plant development and stress responses, in this paper, by measuring 3315 metabolite features in 311 rice varieties, our understanding of natural variation at the metabolite level of rice has largely furthered. More than 39% of the metabolite features identified showed large fold change (> 2) within wild and cultivars in this study, which provided an interesting direction to explore how the huge quantitative variations regulate the growth and development in rice. The results showed that wild rice have a higher sd1825 content compared to cultivars, and behave in purple leaf sheath color. Besides, transgenic plants with lower sd1825 content are sensitive to cold tolerance compared to wild type. Since wild rice grows in the natural environment, which is harsher than that of cultivars, these finding suggest that high sd1825 content in wild rice may play a crucial role in protecting plants against a range of abiotic and biotic stresses.

Anthocyanins have strong anti-mutation and antioxidant activities, and are important to human health (Li et al. 2017a, b), for example, black rice contains high levels of anthocyanins in the pericarp and is considered an effective health-promoting food (Yoshimura et al. 2012). We present a comprehensive study of rice metabolism, combining omics technologies such as genome and transcriptome, and identified that a UDP 3-*O*-glucosyltransferase named *OsLSC6 *which involved in the anthocyanin biosynthesis of Cyanidin-3-Galc (sd1825). *OsLSC6* plays a crucial role in regulating rice leaf sheath color. Knockout of *OsLSC6* resulted in a significant decline of anthocyanins sd1825 content in wild rice and resulted in a green leaf sheath. Meanwhile, anthocyanins also play a key role in resisting biotic and abiotic stresses, such as cold (Schulz et al. 2015, 2016; Li et al. 2017a, b). Our study has demonstrated that *OsLSC6* played a crucial role in regulating rice cold tolerance stress, which is different from other reported stress related UGTs, such as UGT76C2 and UGT71C5 (Li et al. 2015; Liu et al. 2015). Knockout of the *OsLSC6* gene in wild rice accession DX386 showed significantly lower seedling survival rates compared to wild type under cold treatment (Fig. 4), these results demonstrated that *OsLSC6* positively regulates cold stress in rice. This highlights the potential of *OsLSC6*, which involved in the anthocyanin biosynthesis of sd1825, responsible for the purple leaf sheath color and enhanced cold tolerance with higher accumulation of sd1825 in rice.

We sequenced the full-length of *OsLSC6* in 311 rice accessions and identified a nonsynonymous polymorphism in the second exon (Fig. 2E), the evidence showed that the SNP (C798A) in the coding region of *OsLSC6* endows wild rice with purple leaf sheath color. Strikingly, more than 88% (142 of 160) of the wild rice harbored the *OsLSC6*^C^ haplotype performed purple leaf sheath color, whereas 67% (101 of 151) cultivars harbored the *OsLSC6*^A^ haplotype, and performed green leaf sheath color (Fig. 2G and Table S5), this suggests that the SNP, resulting in a change of amino acid in the coding region of *OsLSC6*, may responsible for the purple leaf sheath color in rice.

Our further research revealed that *OsLSC6* also confers resistance to cold stress during the seedling stage in rice. We grouped the 411 cultivated rice accessions (including the 120 cultivars used in this study) based on the phenotype of seedling survival rates and genotype at the *OsLSC6* locus, and examined whether the cold tolerance was associated with the SNP (C798A) in *OsLSC6*. The results showed that the cultivars with the genotype *OsLSC6*^C^ displayed significantly higher survival rates than the cultivars with the genotype *OsLSC6*^A^ after cold treatment. Our data suggest that the SNP make a contribution to seedling chilling tolerance.

## Conclusion

In this study, the identified *Oryza sativa* leaf sheath color 6 (*OsLSC6*) which encoded a UDP 3-*O*-glucosyltransferase is involved in rice anthocyanin biosynthesis and chilling tolerance. And we identify a SNP in the coding region of *OsLSC6* is responsible for the leaf sheath color and cold stress response. Therefore, this study guides for rice breeding to improve rice cold tolerance and high content of anthocyanin.

## Materials and Methods

### Plant Materials

Genetic materials used in this study included 311 rice accessions were analysed including 160 wild rice and 151 cultivars, information about the accessions is listed in Table S1. To study natural variation of the metabolome, the rice leaf samples were randomly collected for metabolic profiling. Leaves at the five-leaf stage were used for metabolic analysis, the samples were obtained between 9:00 and 12:00 am, then placed in liquid nitrogen immediately. The leaf samples were taken from three different plants and pooled together for each biological replicate. The wild rice variety DX386 showed purple leaf sheath with high anthocyanin content (sd1825), was selected as recipient for the *OsLSC6* knockout transgenic test.

### Metabolite Profiling

A relative quantification method of widely targeted metabolites was used to analyze samples (Cao et al. 2022). The samples were crushed using a grinder (MM 400, Retsch, Germany) with zirconia beads for 1.5 min at 30 Hz. Next, about 100 mg of sample powder extracted overnight with 1.0 ml of – 20 °C pre-cooled 70% aqueous methanol internal standard extract at 4 °C. Following centrifugation at 12 000 rpm for 10 min at 4 °C, vortex once every 30 min for 40 s, for a total of 5 times. After centrifugation, the supernatant was aspirated, and the sample was absorbed and filtered through a microporous membrane and stored in the injection vial for UPLC-MS/MS analysis (Chen et al. 2013).

The analytical conditions were as follows, UPLC: column, Agilent SB-C18; The mobile phase was consisted of solvent A, pure water with 0.1% formic acid, and solvent B, acetonitrile with 0.1% formic acid. Sample measurements were performed with a gradient program that employed the starting conditions of 95% A, 5% B. Within 10 min, a linear gradient to 5% A, 95% B was programmed, and a composition of 5% A, 95% B was kept for 1 min. Subsequently, a composition of 95% A, 5% B was adjusted within 1 min and kept for 3 min. The flow velocity was set as 0.35 ml per minute; The column oven was set to 40 °C; The injection volume was 2 μL. The effluent was alternatively connected to an ESI-triple quadrupole-linear ion trap (QTRAP)-MS.

### Population Structure Analyses Using Metabolomics Data

Neighbor-joining tree and PCA plots were used to infer the structure of the 311 rice population. The data matrix was generated from 311 rice varieties and 3315 metabolites, which represented the contents of each metabolite in different populations. Unsupervised PCA was performed with metabolite data by statistics function prcomp within R (www.r-project.org). The data was unit variance scaled to improve normality before unsupervised PCA. Identification of differential accumulation of metabolites between different varieties was determined by partial least squares-discriminate analysis (PLS-DA) with VIP values ≥ 1, followed by ANOVA (P ≤ 0.05).

### Genome‑Wide Association Study

A total of 292,485 SNPs were used for the GWAS. Population structure was modeled by admixture (Alexander et al. 2009), only SNPs with a moderate MAF (minor allele frequency) ≥ 0.05 were employed. mGWAS was performed using the LMM (linear mixed model) implemented in TASSEL (Zhang et al. 2010). The genome-wide significance thresholds was determined using the Bonferroni test threshold (*P* = 2.61E−7), and the lead SNP within the 100-kb window for each metabolite was extracted as one signal. SNP with the lowest *P* value (lead SNP) and its corresponding genes were believed for each significant metabolic site.

### RNA-Sequencing Data Analysis

RNA sequencing was performed use the leaf samples of 15 wild rice with higher sd1825 accumulation showed purple leaf sheath, and 15 cultivars with lower sd1825 content showed green. Total RNA was extracted using trizol reagent (Invitrogen) according to the manufacturer’s protocol. Sequenced clean data were mapped onto the rice reference genome (MSU7) using Hisat2 software with default parameter, and expression level of genes were calculated using StringTie software and the GTF annotation file of MSU7.

### Genotyping of *OsLSC6*

Genome resequencing was performed for the 311 rice accessions used in this study. Total genomic DNA was extracted from leaves using CTAB buffer (Cao et al. 2022). Amplification of *OsLSC6* was performed by PCR using a PCR Mix (2xTSINGKE Master Mix, TSE004). PCR was performed in a Bio-Rad thermo cycler T100 (ThermoFisher SCIENTIFIC) with the following cycling profile: 95 °C for 5 min, followed by 35 cycles of 95 °C for 30 s, 56 °C for 30 s, and 72 °C for 1 min, and a final 10 min extension at 72 °C, the primers used to amplify the *OsLSC6* are list in Table S7.

### Vector Construction and Rice Transformation

To produce the *OsLSC6* gene editing vector using CRISPR/Cas9 technology, we selected two target sites in this study (GGGAACATGAGGTTCGTGGAGGTCG and GAACACCGCCATCCGCCGCGGCGG). Subsequently, the gene editing vectors were transformed into the wild rice DX386, which has high sd1825 content, and the transformation was achieved using the *Agrobacterium*-mediated method.

### Cold Treatment

To test the rice cold tolerance, the seedlings were treated at 10 °C for at least 13 days, subsequently, they were moved to artificial climate chamber with 28 °C/26 °C day/night cycles for recovery. After 10 days, the rice cold tolerance was determined by the percentage of the total rice seedlings survival rate (Ma et al. 2009).

### Supplementary Information

Supplementary Material 1: Table S1. The list of 160 wild and 151 cultivars used in this study. Table S2. Comparing levels of metabolites in the wild and cultivars. Table S3. The CVs of the metabolic features in wild and cultivars. Table S4. All the 101 annotated flavonoids detected in this study. Table S5. The genotype of all the 311 wild and cultivars. Table S6. The seedling survival rates of 411 rice accessions. Table S7. The primers used to amplify *OsLSC6*.

Supplementary Material 2: Figure S1: The number of different classes of metabolic features compared wild rice to cultivars (A), distribution of the genetic coefficients of variation (CV) of metabolic traits (n = 3315) in wild and cultivars (B), distribution of broad-sense heritability (*H*^*2*^) of metabolic traits detected in the metabolite panel across two biological replicates (C), neighbor-joining tree of 311 rice accessions, which was calculated from 292,485 SNPs, identifies the two groups of wild and cultivars (D).

Supplementary Material 3: Figure S2. Principal component analysis of 311 rice accessions according to their metabolome profiles.

Supplementary Material 4: Figure S3: Manhattan plot displaying the GWAS result of the phenotype of leaf sheath color.

Supplementary Material 5: Figure S4: The MS spectrums and chemical structure of Cyanidin-3-Glac (sd1825) (A), boxplot showing the content of sd1825 in wild and cultivars (B).

## Data Availability

All data supporting the conclusions of this article are provided within the article (and its additional files).

## Abbreviations

PCA: Principal component analysis
VIP: Variable importance in the projection
FC: Fold change
CV: Coefficient of variation
MAF: Minor allele frequency
LMM: Linear mixed model
WT: Wild type
EBG: Early biosynthetic genes
LBG: Late biosynthetic genes
