# Supplementary figures and images for: OsLSC6 Regulates Leaf Sheath Color and Cold Tolerance in Rice Revealed by Metabolite Genome Wide Association Study
Source: Rice (N Y). 2024 May 13;17:34. doi: 10.1186/s12284-024-00713-z (PMC11091021; doi:10.1186/s12284-024-00713-z)

A

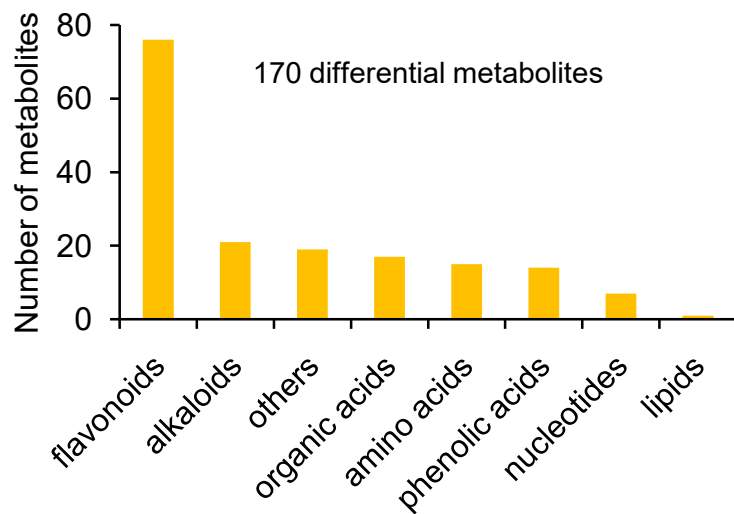

B

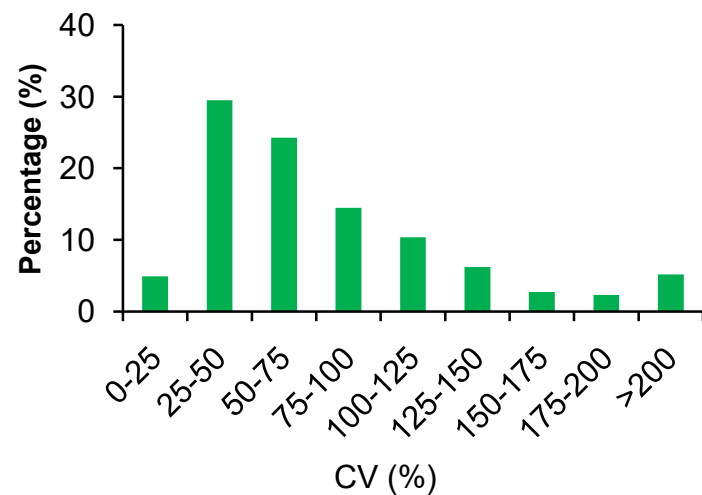

C

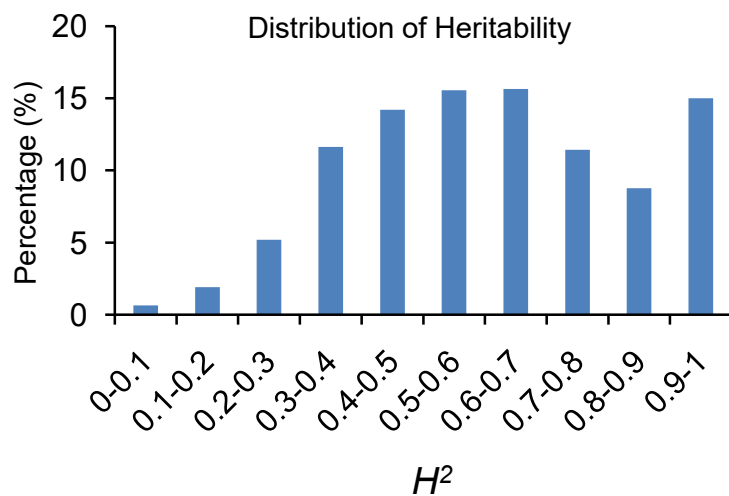

D

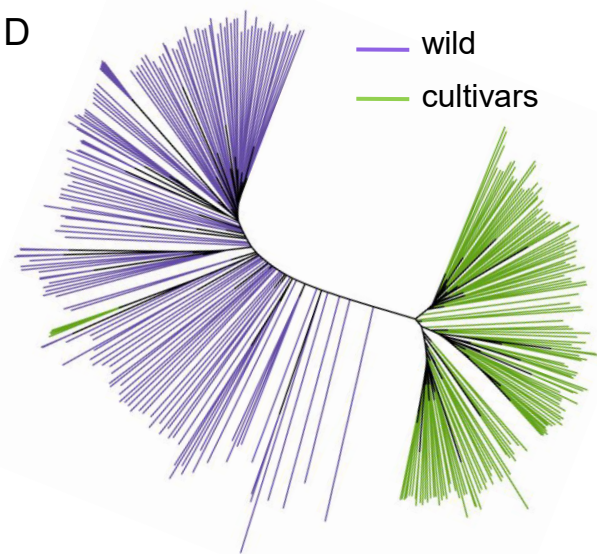

Supplement: Supplementary file 2 — Supplementary Material 2: Figure S1: The number of different classes of metabolic features compared wild rice to cultivars (A), distribution of the genetic coefficients of variation (CV) of metabolic traits (n = 3315) in wild and cultivars (B), distribution of broad-sense heritability (H2) of metabolic traits detected in the metabolite panel across two biological replicates (C), neighbor-joining tree of 311 rice accessions, which was calculated from 292,485 SNPs, identifies the two groups of wild and cultivars (D). [file 12284_2024_713_MOESM2_ESM.pdf]

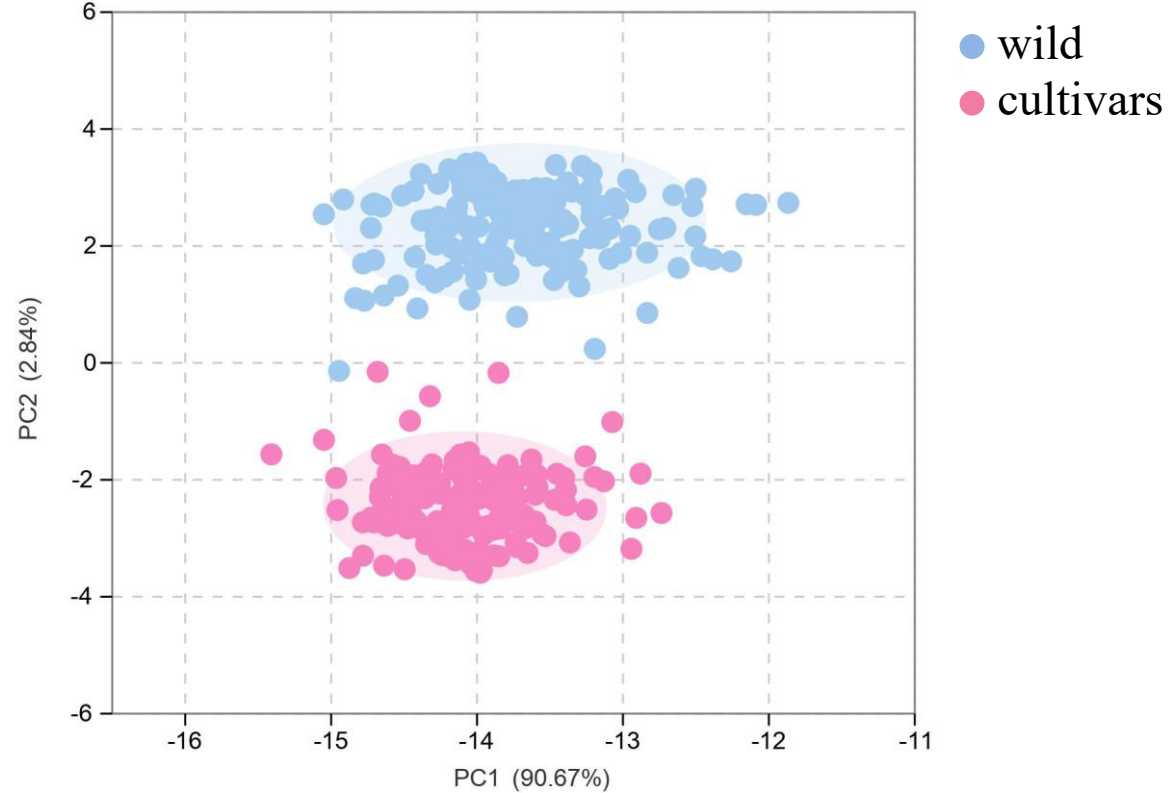

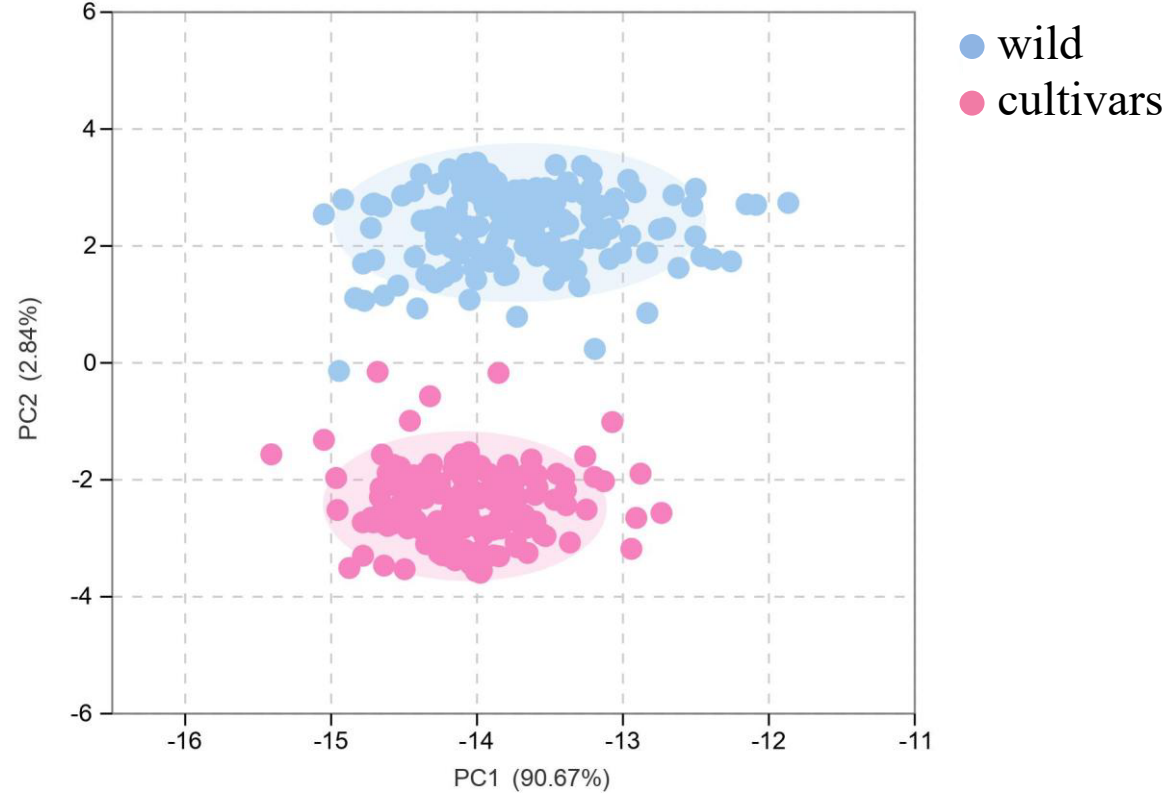

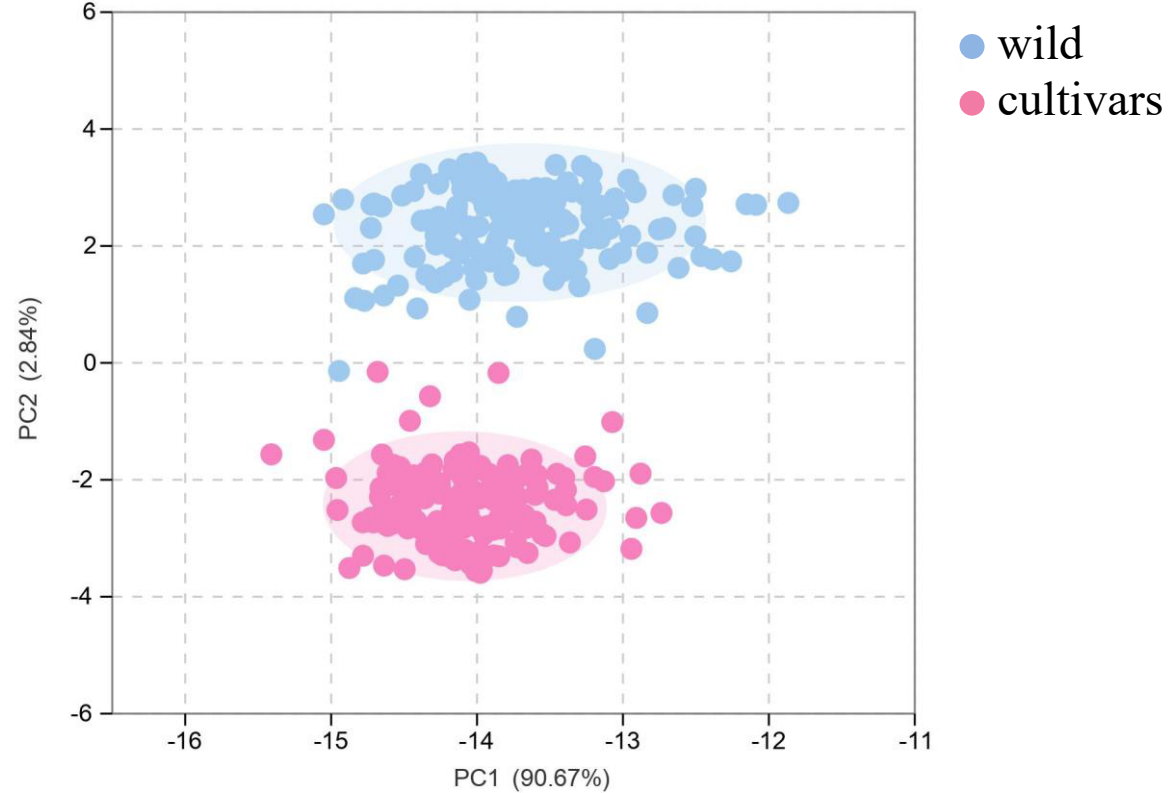

Supplement: Supplementary file 3 — Supplementary Material 3: Figure S2. Principal component analysis of 311 rice accessions according to their metabolome profiles. [file 12284_2024_713_MOESM3_ESM.pdf]

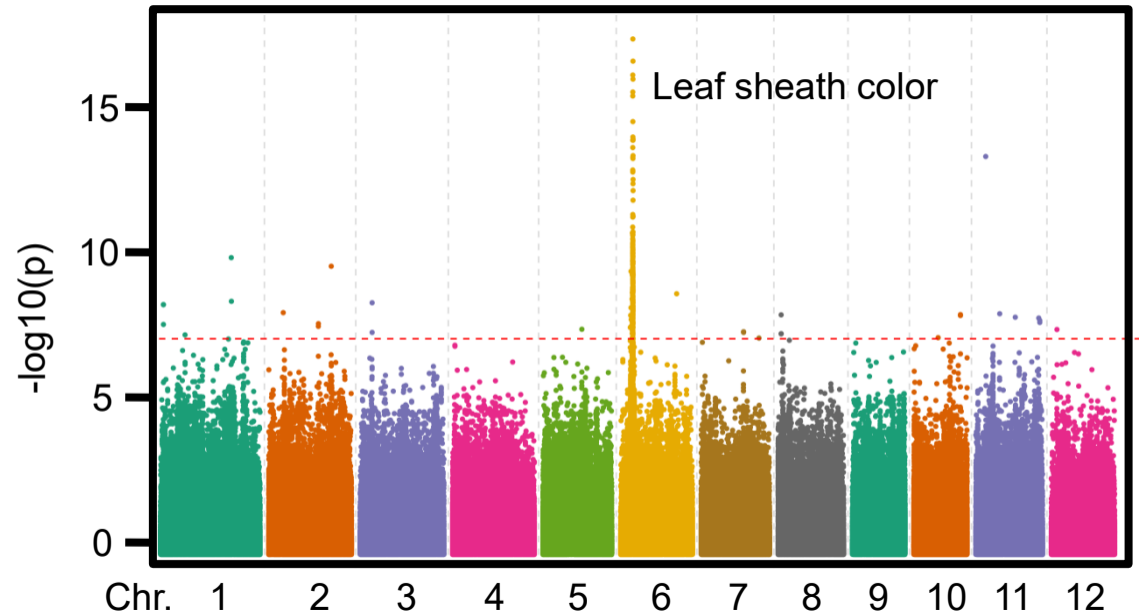

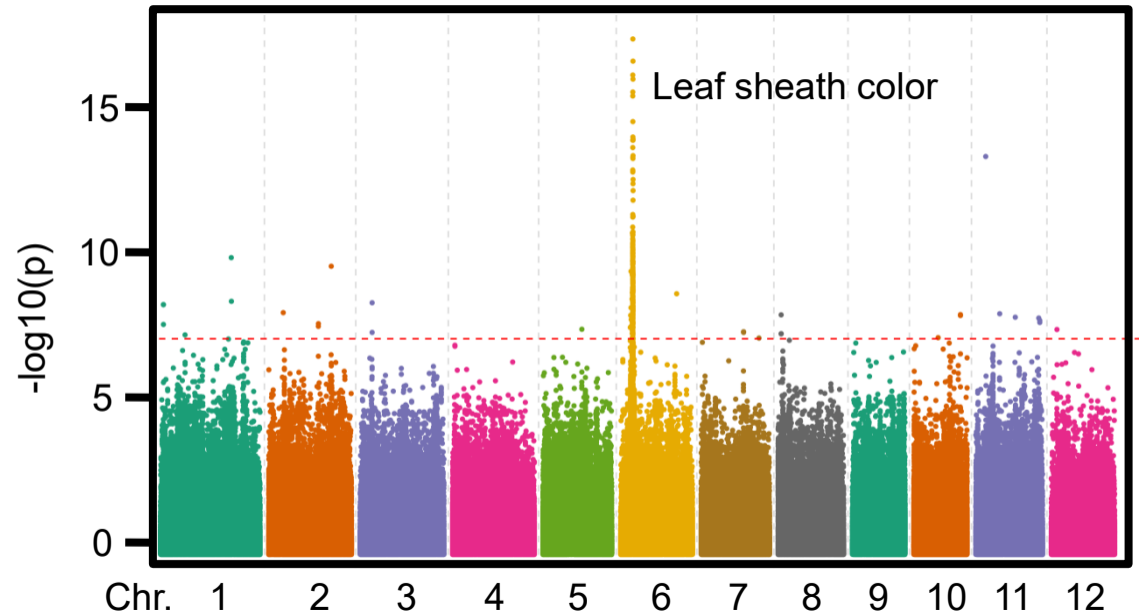

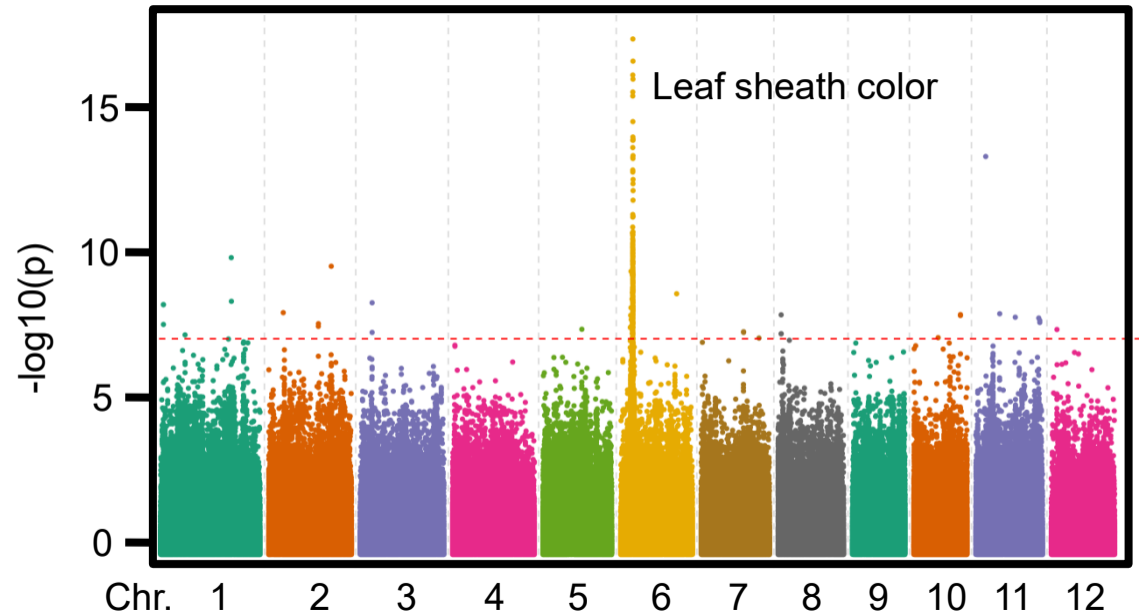

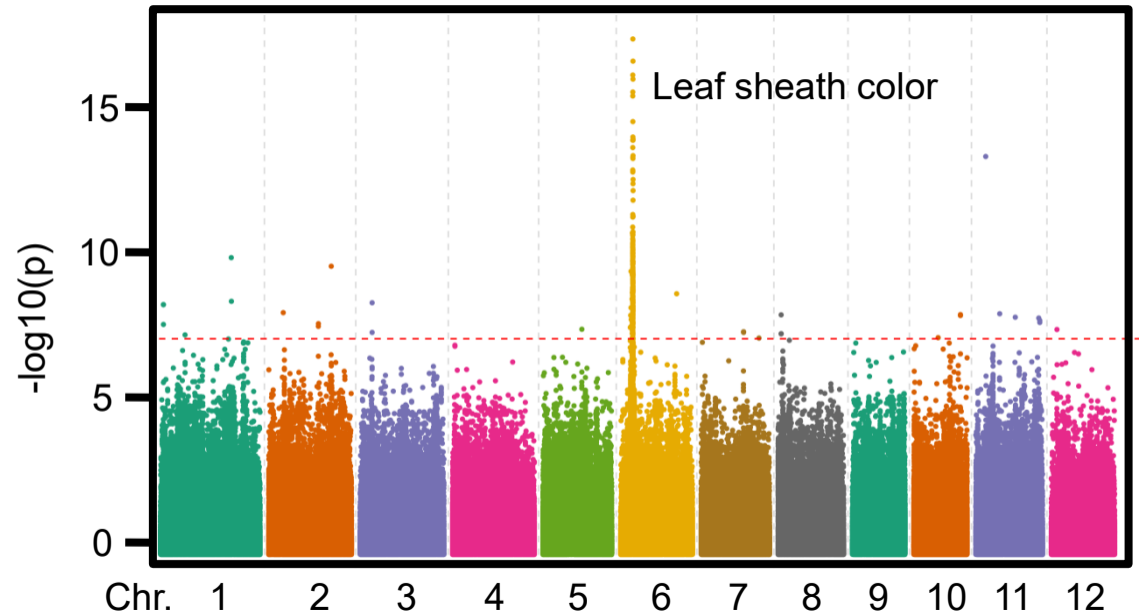

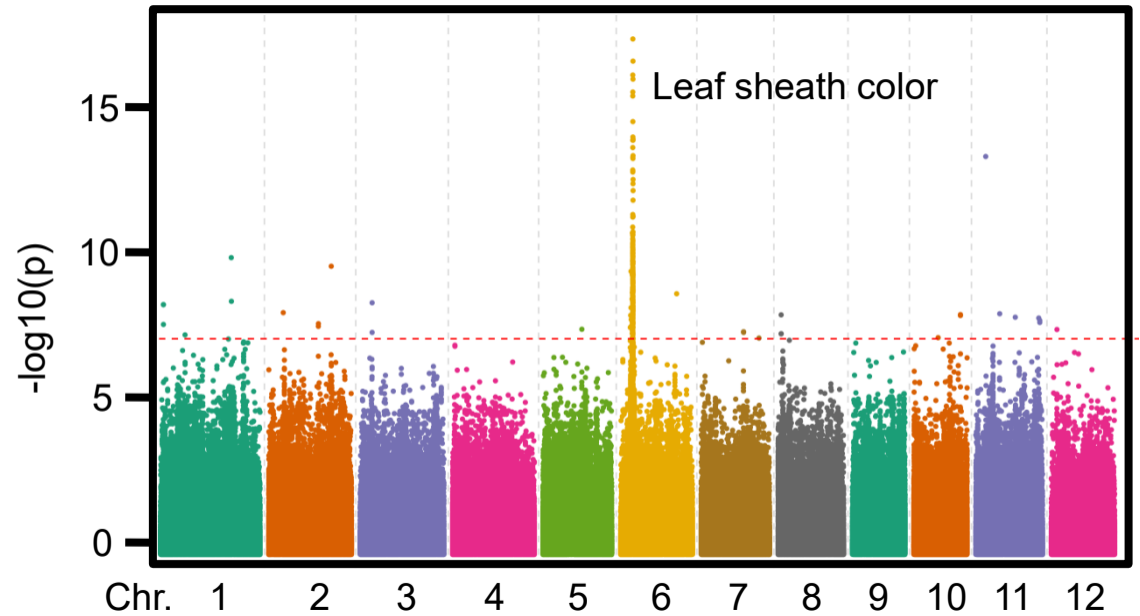

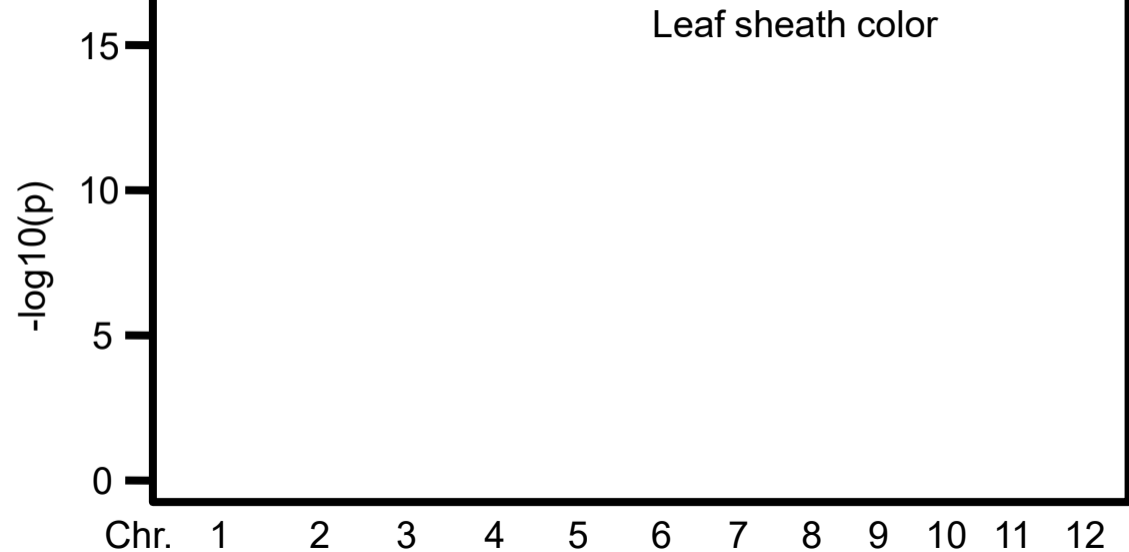

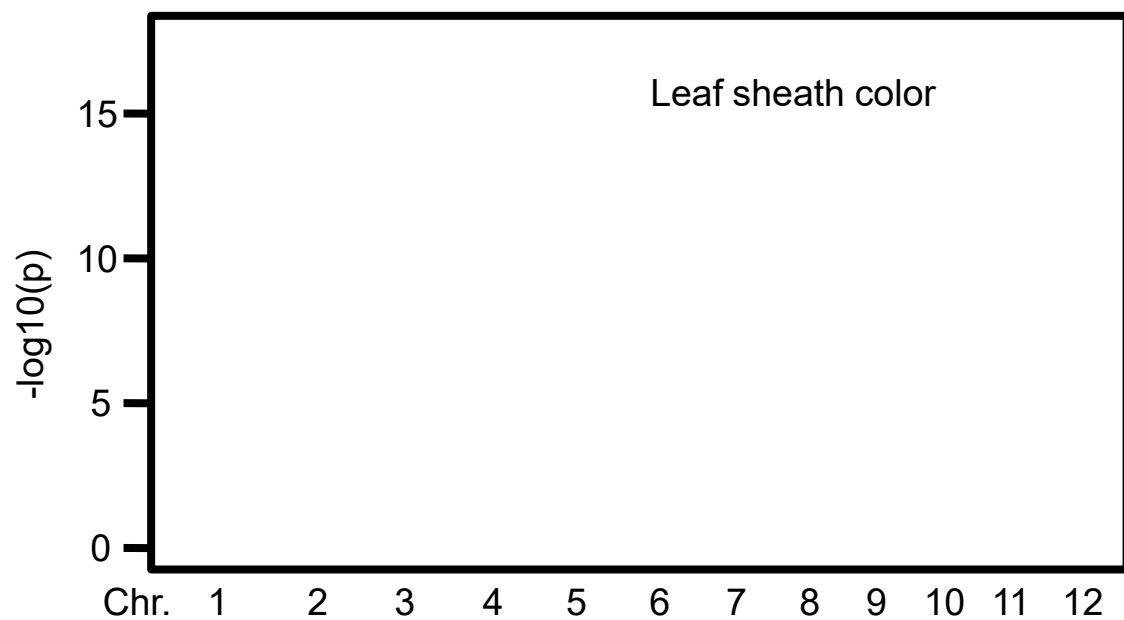

Supplement: Supplementary file 4 — Supplementary Material 4: Figure S3: Manhattan plot displaying the GWAS result of the phenotype of leaf sheath color. [file 12284_2024_713_MOESM4_ESM.pdf]

# Cyanidin-3-Gluc

A

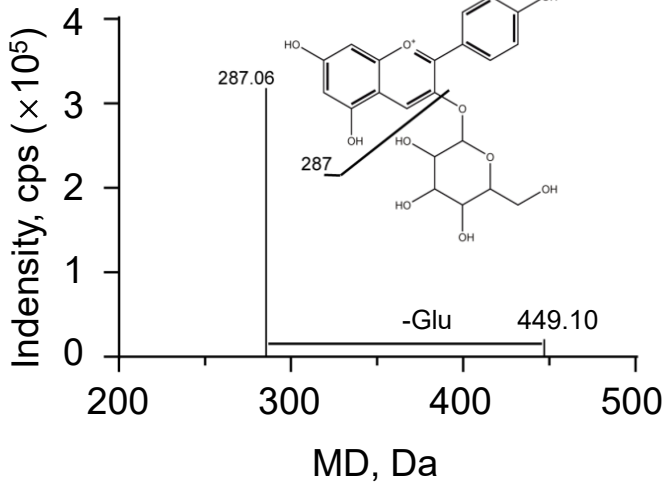

Structure of Cyanidin-3-Gluc

B

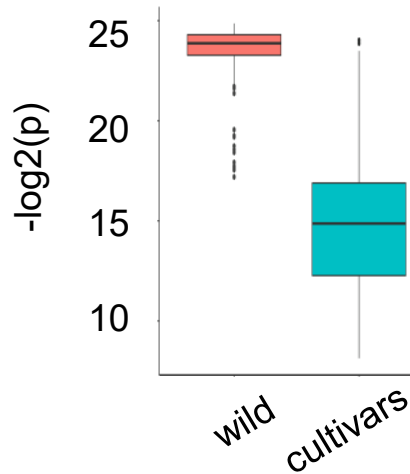

Supplement: Supplementary file 5 — Supplementary Material 5: Figure S4: The MS spectrums and chemical structure of Cyanidin-3-Glac (sd1825) (A), boxplot showing the content of sd1825 in wild and cultivars (B). [file 12284_2024_713_MOESM5_ESM.pdf]
